# Enhancing multi-class neurodegenerative disease classification using deep learning and explainable local interpretable model-agnostic explanations

**DOI:** 10.3389/fmed.2025.1562629

**Published:** 2025-04-01

**Authors:** Jamel Baili, Abdullah Alqahtani, Ahmad Almadhor, Abdullah Al Hejaili, Tai-hoon Kim

**Affiliations:** ^1^Department of Computer Engineering, College of Computer Science, King Khalid University, Abha, Saudi Arabia; ^2^Department of Computer Science, College of Computer Engineering and Sciences, Prince Sattam bin Abdulaziz University, Al-Kharj, Saudi Arabia; ^3^Department of Computer Engineering and Networks, College of Computer and Information Sciences, Jouf University, Sakaka, Saudi Arabia; ^4^Faculty of Computers and Information Technology, Computer Science Department, University of Tabuk, Tabuk, Saudi Arabia; ^5^School of Electrical and Computer Engineering, Chonnam National University, Yeosu-si, Jeollanam-do, Republic of Korea

**Keywords:** deep learning models, Parkinson's disease (PD), Alzheimer's disease (AD), neurodegenerative disorders, medical image analysis

## Abstract

**Introduction:**

Alzheimer's disease (AD) and Parkinson's disease (PD) are two of the most prevalent neurodegenerative disorders, necessitating accurate diagnostic approaches for early detection and effective management.

**Methods:**

This study introduces two deep learning architectures, the Residual-based Attention Convolutional Neural Network (RbACNN) and the Inverted Residual-based Attention Convolutional Neural Network (IRbACNN), designed to enhance medical image classification for AD and PD diagnosis. By integrating self-attention mechanisms, these models improve feature extraction, enhance interpretability, and address the limitations of traditional deep learning methods. Additionally, explainable AI (XAI) techniques are incorporated to provide model transparency and improve clinical trust in automated diagnoses. Preprocessing steps such as histogram equalization and batch creation are applied to optimize image quality and balance the dataset.

**Results:**

The proposed models achieved an outstanding classification accuracy of 99.92%.

**Discussion:**

The results demonstrate that these architectures, in combination with XAI, facilitate early and precise diagnosis, thereby contributing to reducing the global burden of neurodegenerative diseases.

## 1 Introduction

Neurological disorders, including Alzheimer's Disease and Parkinson's Disease, pose significant health concerns, affecting millions of people worldwide each year (1). AD is a leading cause of progressive, irreversible dementia worldwide, accounting for 60–80% of cases (2), whereas PD affects 2–3% of adults over 60, making it the second most common neurodegenerative disorder. Its prevalence has doubled in the past 25 years, leading to increased disability and mortality (3). Dementia is a continuum of neurological conditions characterized by memory loss, impaired reasoning, and difficulty performing daily activities (4). According to the World Alzheimer's Report, over 55 million people worldwide have dementia, with projections indicating that this number will rise to 152 million by 2050. This growing prevalence underscores the urgent need for early diagnosis and preventive measures (5).

A key pathological hallmark of AD is the accumulation of amyloid-beta (Aβ) plaques and neurofibrillary tangles of tau protein, which disrupt synaptic communication and trigger neuronal degeneration (6). Many of these changes occur years before clinical symptoms appear. Mild Cognitive Impairment (MCI) is an intermediate stage between normal aging and dementia, with 10-30% of MCI patients progressing to AD annually (7). Early identification of MCI is crucial, as individuals may benefit from medical interventions that slow the progression of dementia. Similarly, PD, which initially presents with motor symptoms such as tremors, rigidity, and bradykinesia, can advance to Parkinson's Disease Dementia (PDD), complicating patient care and treatment strategies (8).

Recent advancements in imaging technologies, including MRI, PET, and CT, have significantly improved the diagnosis of neurodegenerative diseases (9). These modalities enable the tracking of structural and functional brain alterations associated with AD and PD. However, conventional medical image analysis is time-consuming and susceptible to human bias, highlighting the need for automated and accurate diagnostic tools (10).

Artificial intelligence (AI), particularly deep learning (DL), has emerged as a powerful tool for medical image classification and pattern recognition (11). CNNs have demonstrated exceptional performance in analyzing medical images, identifying subtle biomarkers that human clinicians may overlook (12). However, traditional DL models face challenges such as the “black box” problem and lack of interpretability, limiting their acceptance in clinical settings (13). To address these limitations, advanced architectures like Residual Self-Attention CNNs and Inverted Residual Self-Attention CNNs have been developed. These models enhance feature extraction, improve interpretability, and achieve higher accuracy across diverse datasets.

This study explores the feasibility of using RbACNN and IRbACNN models for early-stage AD and PD classification from medical images. By leveraging these advanced deep learning techniques, the research aims to overcome challenges such as poor image quality, variability across patients, and the complex structure of brain regions (14). Additionally, the study utilizes publicly available datasets like ADNI and OASIS, along with preprocessing tools such as SPM and Freesurfer, to ensure reliable and reproducible results. Ultimately, this work seeks to enhance early diagnosis, improve patient outcomes, and contribute to reducing the global burden of neurodegenerative diseases.

The three main contributions of this study are given below:

- This study introduces enhanced deep learning architectures, Residual-based Attention Model and Inverted Residual-based Attention Model, combined with explainable AI techniques to improve the interpretability of multi-class classification for Alzheimer's and Parkinson's diseases. This enhances both diagnostic accuracy and clinical transparency.
- The proposed models leverage advanced self-attention mechanisms to improve feature extraction and classification performance. In addition, comprehensive preprocessing techniques, including histogram equalization and batch creation, were employed to enhance image quality and ensure a balanced dataset for robust model training.
- The models achieved remarkable classification accuracies of up to 99.92%, demonstrating their effectiveness in distinguishing between Alzheimer's disease, Parkinson's disease, and healthy control groups. The integration of XAI further supports their applicability in clinical decision-making.

This study is organized into sections to clarify the research framework. Section 2 highlights the related work addressing Alzheimer's disease detection by using DL models. The proposed methods, dataset details, preprocessing strategies, and discussions on proposed models are elaborated in Section 3. Section 4 focuses on evaluation metrics, experimental findings, and conclusions, including suggestions for future research. Table 1 provides the summary of the abbreviation used in the paper.

**Table 1:** List of abbreviations and their descriptions.

**Abbreviation**	**Description**
AD	Alzheimer's disease
PD	Parkinson's disease
MCI	Mild cognitive impairment
PDD	Parkinson's disease dementia
MRI	Magnetic resonance imaging
PET	Positron emission tomography
CT	Computed tomography
AI	Artificial intelligence
DL	Deep learning
CNN	Convolutional neural network
SPM	Statistical parametric mapping
PCA	Principal component analysis
DNN	Deep neural network
KNN	K-nearest neighbors
RF	Random forest
SVM	Support vector machine
XGBoost	Extreme gradient boosting
AUC	Area under the curve
ROC	Receiver operating characteristic
MSE	Mean squared error

## 2 Related work

This Section explains the related work of deep learning and advanced machine learning (ML) for neurodegenerative disorders and enhancing diagnostic accuracy. Studies on Alzheimer's disease prediction are summarized in Table 2.

**Table 2:** Summary of related work on Alzheimer's disease classification.

**References**	**Method/approach**	**Dataset**	**Performance metrics**
Yedavalli and Bair (15)	Enhanced CNN with ReLU activation, batch normalization, and dropout	6,400 MRI images (balanced)	Training accuracy: 99.7%, Testing accuracy: 88.79%
Rao et al. (16)	3D CNN with ResNet50V2, transfer learning, and fine-tuning	Not specified	Training accuracy: 92.15%, Testing accuracy: 91.25%
Srividhya et al. (17)	Pre-trained models (ResNet50V2), visualization with Grad-CAM and Saliency Map	ADNI2 dataset	Accuracy: 91.84%, F1-score: 0.97
Shinde et al. (18)	GLCM feature extraction, fine-tuned pre-trained models, and CNN	Kaggle Dataset	Comparative analysis, performance varies
Hazarika et al. (19)	DNN with dense and inception blocks, PCA for dimensionality reduction	NA	Accuracy: 98.08%
Biswas and Gini J (20)	Hippocampal, white matter, and gray matter volume segmentation, Random Forest	OASIS, ADNI datasets	Accuracy: 99% (OASIS), 92% (ADNI)
Tripathi and Kumar (21)	Speech-based classification with XGBoost and acoustic feature extraction	DementiaBank's Pitt Corpus	Accuracy: 75.59%
Ghassan Al Rahbani et al. (25)	ResNet and EfficientNet ensemble with post-processing	ADNI, OASIS datasets	Accuracy: 98.97% (ADNI), 99.41% (OASIS)
Yao et al. (22)	AD_Net with CBAM and MLP for multi-factor integration	ADNI dataset	Accuracy: 89% (with directional factors)

Yedavalli and Bair (15) proposed an enhanced CNN model for classifying MRI images into four stages of Alzheimer's disease: non-demented, very mildly demented, mildly demented, and moderately demented. The model consisted of four convolutional layers with ReLU activation, batch normalization, and max-pooling, followed by fully connected layers with dropout regularization to prevent overfitting. Trained on a balanced dataset of 6,400 MRI images, the model achieved a peak training accuracy of 99.7% and a testing accuracy of 88.79% on unseen data, demonstrating its effectiveness for accurate AD stage classification. Rao et al. (16) proposed a novel deep-learning framework for Alzheimer's disease classification. Recognizing the limitations of manual classification methods, they leveraged 3D convolutional neural networks to process spatial information within 3D MRI scans. They used differential weights extracted from various layers and integrated transfer learning with the fine-tuning concept, which produced high accuracy. In particular, ResNet50V2 was the chosen best pre-trained model that yielded a training accuracy of 92.15% and a test accuracy of 91.25%. These results demonstrate the feasibility of using transfer learning and deep learning techniques for AD diagnosis. Srividhya et al. (17) employed pre-trained models on the Alzheimer's Disease Neuroimaging Initiative (ADNI2) dataset to differentiate AD stages. They compared existing deep learning algorithms for multi-class classification of MRI images, identifying ResNet50V2 as the most accurate model with a total accuracy of 91.84% for the AD class and an F1-score of 0.97. Grad-CAM and Saliency Map visualization methodologies were used to clarify which regions the model focused on when predicting various stages of illness.

Shinde et al. (18) extracted texture features using the GLCM from MRI images and employed these features to train KNN, Random Forest, and Decision Tree models. Additionally, they studied transfer learning models, including Xception, DenseNet-121, and ResNet50, and developed a CNN model for direct feature learning. Their experiments compared the effectiveness of these models and techniques for AD prediction. Hazarika et al. (19) proposed a deep neural network (DNN) model leveraging dense blocks inspired by VGG-19 for optimal feature extraction. Incorporating inception blocks and min-max-pooling layers, the network preserved both maximum and minimum valued features. They also employed principal component analysis (PCA) for dimensionality reduction and Random Forest for classification. The proposed method achieved a classification accuracy of 98.08%, particularly in distinguishing CN from EAD. Biswas and Gini J (20) designed a multi-class classification system for early AD detection. They segmented hippocampal, white matter, and gray matter volumes from 3D MRI scans and calculated their respective volumes using Analyze Direct and ITK Snap software. These features, combined with demographic data like age and gender, were used to train ML classifiers such as Random Forest, Gradient Boost, Decision Tree, and KNN. The Random Forest classifier achieved the highest accuracy of 99% with the OASIS dataset and 92% accuracy for the ADNI dataset using Gradient Boost. Tripathi and Kumar (21) introduced a speech-based classification system for cognitive impairments, including AD, MCI, and vascular dementia. Speech data from DementiaBank's Pitt Corpus was preprocessed to extract acoustic features, which were then used to train ML models such as KNN, DT, SVM, XGBoost, and RF. Their approach achieved an accuracy of 75.59% for six-class classification, with XGBoost demonstrating statistically significant superiority over most other models, offering a non-invasive and accessible cognitive impairment diagnostic method.

Yao et al. (22) proposed a multi-class classification and prediction model of AD from MRI images based on deep learning. The proposed model, ADNET, is an augmentation of the conventional VGG19 architecture, incorporating a Convolutional Block Attention Module for improved feature extraction. Using the ADNI dataset, the model classified AD, MCI, and CN conditions. A novel MLP-based model, incorporating additional factors such as age, gender, and Minimal State Examination, achieved a prediction accuracy of 51.2% without directional conditions and 89% with them. Sorour et al. (23) introduced a deep learning algorithm (AD-DL) for screening Alzheimer's disease from brain MRI images. Their framework included preprocessing, model training, and evaluation phases, incorporating five deep learning models for binary classification. Among these, the CNN-LSTM model achieved the best results with an accuracy of 99.92%, highlighting the potential of deep learning for AD diagnosis. Krishna et al. (24) proposed an algorithm derived from particle swarm optimization to optimize the hyperparameters of CNN structures for AD classification. Their model, utilizing lightweight features at the convolution layer, achieved a classification accuracy of 99.53% and an F1-score of 99.63%, demonstrating its potential for improving diagnostic efficiency while reducing clinicians' workload. Ghassan Al Rahbani et al. (25) introduced a novel approach to AD diagnosis using ResNet and EfficientNet CNN models with a unique post-processing module. Their methodology employed a weighted average ensemble learning technique to combine the strengths of both models. The approach was tested on the ADNI and OASIS datasets, achieving top-1 classification accuracies of 98.59% with EfficientNet and 94.59% with ResNet on the ADNI dataset, and 99.36% with ResNet and 99.4% after calibration on the OASIS dataset. These results highlight the effectiveness of ensemble learning in improving diagnostic accuracy. Table 2 provides the summary of related work.

## 3 Proposed framework

**Figure 1:**
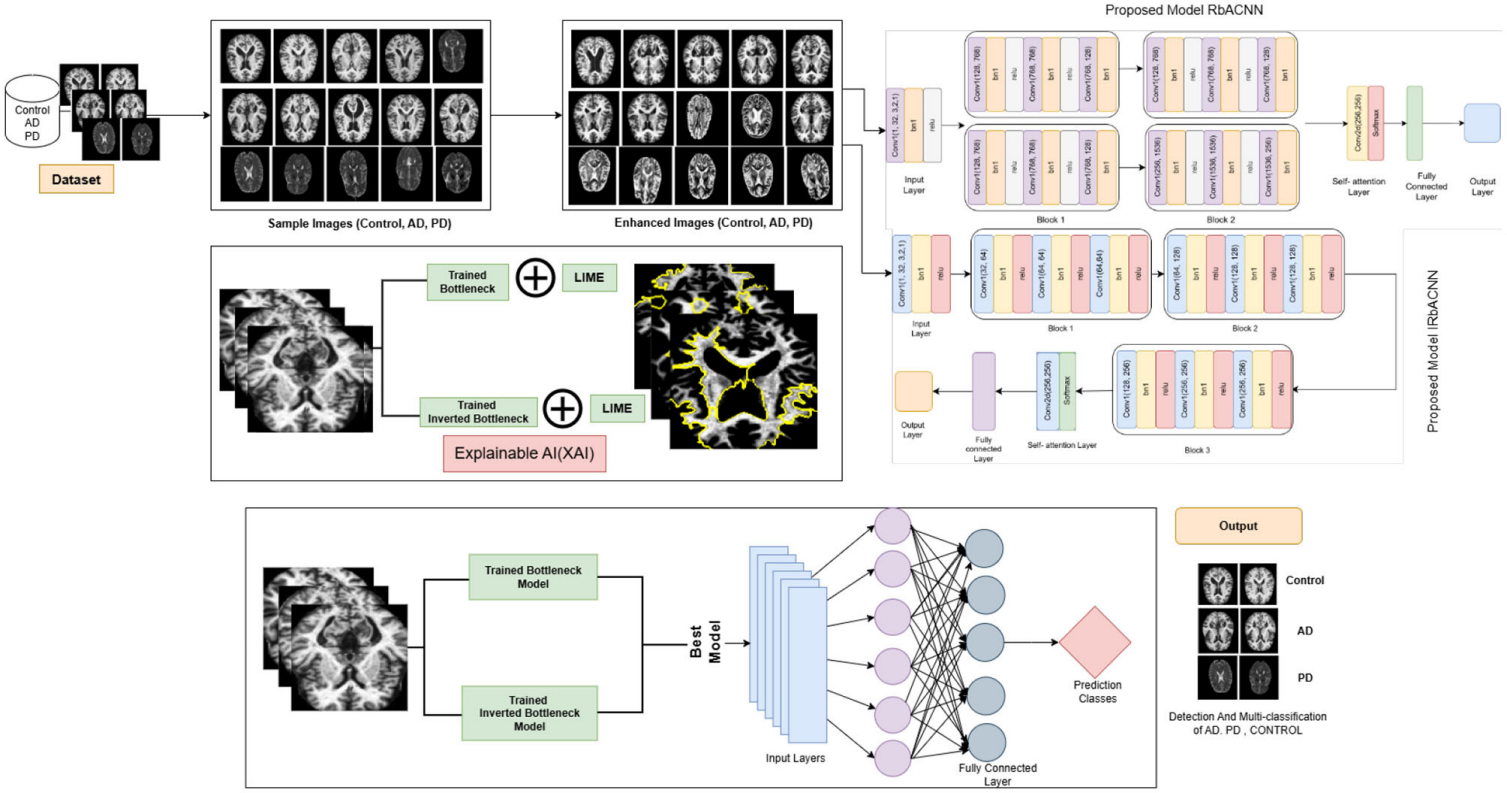
Proposed framework for disease classification.

This Section explains the proposed framework by utilizing deep learning models to identify neurodegenerative diseases. In the classification of this Figure 1 presents a detailed view of the proposed framework, including dataset, image preprocessing and deep learning models.

### 3.1 Experimental dataset and preliminaries

The Alzheimer's Parkinson's Diseases 3 Class dataset is used in this study. The 3_cls folder contains two main directories: train and test. Each of these directories is further divided into three subdirectories: CONTROL, AD, and PD. The CONTROL folder represents normal cases, while AD corresponds to Alzheimer's disease and PD corresponds to Parkinson's disease. This dataset is structured for a three-class classification task involving Alzheimer's, Parkinson's, and control subjects. The number of data files in each category was determined using the Python os module. This step ensures the data is correctly structured and helps to understand the distribution of data across the three groups, which is crucial for downstream analysis. To facilitate testing and validation of image processing techniques, a small batch of images was extracted from the dataset using the create_batch function. This function iterates through the class directories (AD, CONTROL, PD) and selects a specified number of images (up to 800 per class) to create a manageable subset for testing. The selected images are copied into corresponding subdirectories within a new output directory. The batch creation process was logged for debugging purposes, and the final structure of the output directory was verified to ensure proper organization. The contents of the batch directory were confirmed to include the expected class subdirectories AD, CONTROL, and PD.

### 3.2 Image preprocessing and enhancement for dataset optimization

In this Section of the paper, we describe the process of preprocessing and enhancing the images from the dataset to improve their quality for further analysis. The following steps were implemented to ensure that the data is properly prepared for testing and model development:

#### 3.2.1 Image preprocessing

To facilitate efficient processing, the preprocessed dataset with progress function was implemented, which applies a basic image enhancement technique to each image in the dataset. The function processes images from class-specific directories (e.g., AD, CONTROL, PD) and applies histogram equalization to improve the contrast of the grayscale images. The progress of the image processing is tracked and displayed using the progress bar, which provides real-time feedback on the processing status. The enhanced images are saved in a new directory, ensuring that the original data remains intact for future reference. Before applying the enhancement process, a batch of images was created from the dataset using the create batch function. This step selects a fixed number of images (800 images per class) from each class and saves them into a new output directory. The batch creation process ensures that only a manageable subset of the data is processed for testing purposes.

#### 3.2.2 Enhanced images representation

**Figure 2:**
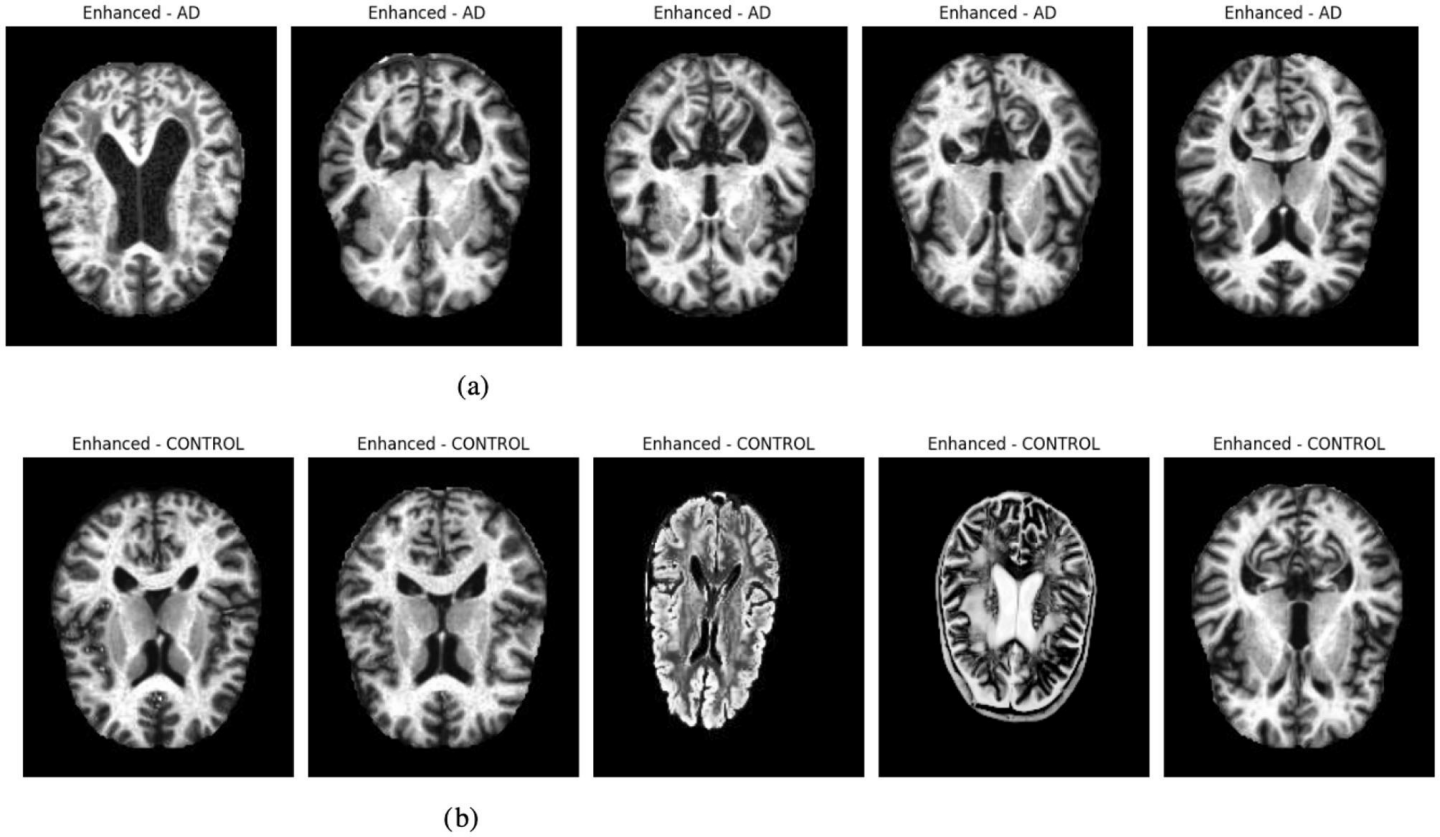
Graphical visualization of original and enhanced image. **(a)** Original and enhanced images of AD label. **(b)** Original and enhanced images of control label.

Figure 2 presents enhanced images for a selection of five samples per class, illustrating the effect of histogram equalization. This enhancement improves contrast and highlights critical structural details, aiding in better feature extraction for deep-learning models. Improved contrast ensures clearer visualization of tissue boundaries and anomalies, which is crucial for accurate classification. The visualization step also helps verify that preprocessing enhances image quality without introducing distortions, ensuring reliable model performance.

### 3.3 Residual based attention CNN model

**Figure 3:**
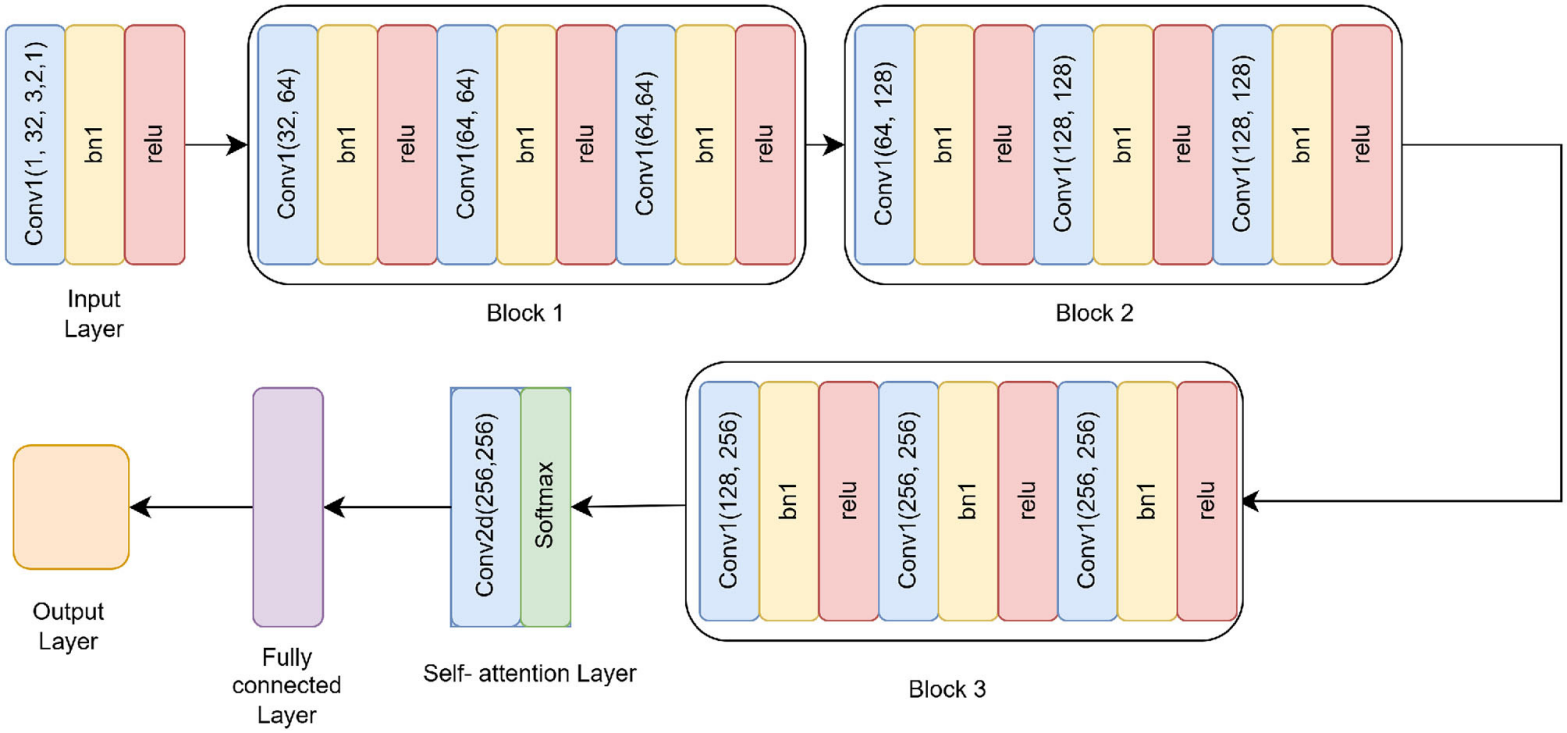
Residual self-attention CNN model architecture.

The Residual-based Attention Model (RbACNN) model is an architecture designed for image classification tasks. It leverages the power of residual blocks and self-attention mechanisms to efficiently extract features and enhance spatial dependencies in the input data (see Figure 3). The model is designed to adapt to the input size dynamically, making it scalable for various datasets (26).

#### 3.3.1 Input layer and initial convolutional

The RbACNN model employs three residual blocks, each designed to expand the feature channels and enhance the model's ability to capture hierarchical feature representations. These blocks share a consistent structure, consisting of convolutional layers interleaved with batch normalization and ReLU activation. However, they differ in their input and output channel dimensions, progressively increasing the depth of feature representations across the network.

**Block 1:** The first block begins by expanding the input channels from 32 to 64. It includes three sequential convolutional layers, each with a kernel size of 3*x*3. Batch normalization is used to normalize the feature activations following each convolution operation, stabilizing the training process. A ReLU activation function comes next, adding non-linearity and enabling the model to recognize intricate patterns in the input data. The repeated application of these layers ensures that the model captures richer features while preserving the spatial dimensions of the input.

**Block 2:** In the second block, the feature channels are further expanded from 64 to 128. While the structure remains identical to the first block, the increase in channel dimensions allows the model to process more complex features. This progression enables the network to extract intermediate-level representations, bridging the gap between initial low-level features and higher-level abstractions found in the later stages of the network.

**Block 3:** The third block completes the feature expansion by increasing the channels from 128 to 256. Following the same design principles as the previous blocks, this stage is critical for learning highly abstract and fine-grained features. The increased channel count at this stage significantly enhances the model's ability to discriminate between subtle variations in the data, making it well-suited for complex classification tasks.

#### 3.3.2 Self-attention mechanism

To enhance spatial feature dependencies, the RbACNN incorporates a self-attention mechanism. This mechanism applies a 1 × 1 convolution to reduce dimensionality, followed by a softmax activation function along the spatial dimensions. The self-attention layer computes an importance map, which is element-wise multiplied by the feature map, emphasizing critical regions of the input data. This step is crucial for improving the model's focus on discriminative features.

#### 3.3.3 Dynamic fully connected layer

One unique aspect of the RbACNN model is its dynamically initialized fully connected (FC) layer. After the self-attention mechanism, the feature map is flattened into a 1D vector. The FC layer is instantiated during runtime based on the input feature size, ensuring compatibility with any input image dimensions. This adaptability enhances the model's usability across diverse datasets.

#### 3.3.4 Model training and optimization

The RbACNN model is trained to classify input data into a predefined number of classes. The FC layer's output neuron count corresponds to the number of classes. The model employs cross-entropy loss function and optimization techniques such as Adam to ensure efficient convergence during training. The model is initialized with the number of classes derived from the dataset's class map. The architecture of the model represented as RbACNN, is specific to the task at hand. The code ensures compatibility with the hardware by moving the model to a GPU if available or to a CPU otherwise. This setup ensures efficient computation during training and inference. Before beginning the training process, this Section performs a quick forward pass using a batch of images to verify the model's functionality. Images and labels are loaded from the batch_loader and moved to the selected device. The model processes the images to produce outputs, ensuring no runtime errors occur during the forward pass. The CrossEntropyLoss function, which works well for multi-class classification applications, is used in the training pipeline. With an initial learning rate of 0.001, the Adam optimizer is selected due to its flexible learning rate capabilities. These choices ensure stable and efficient optimization of the model's parameters.

#### 3.3.5 Explainability with LIME

**Figure 4:**
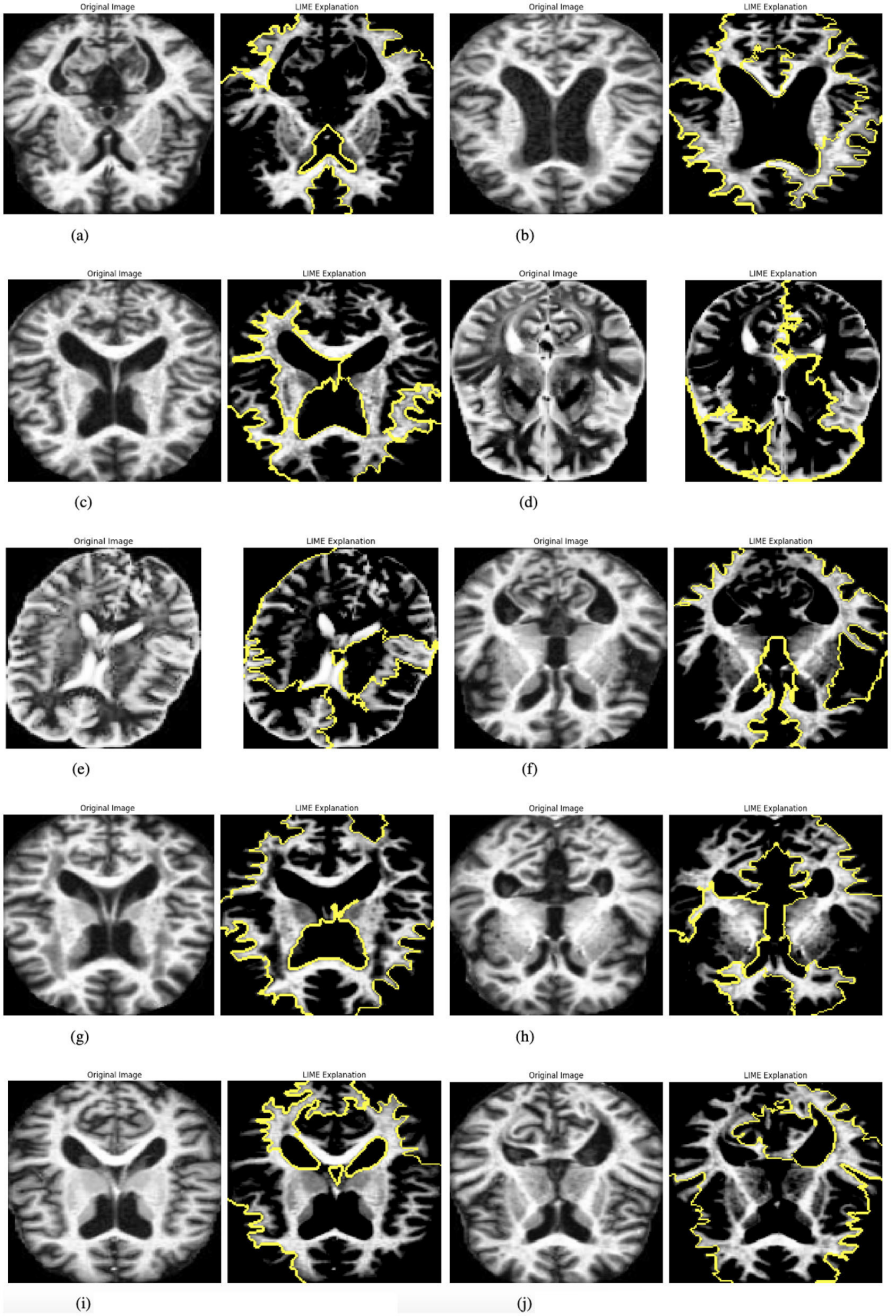
Graphical visualization of original image and explainability LIME of RbACNN model. **(a)** Original image and LIME explanation 1. **(b)** Original image and LIME explanation 2. **(c)** Original image and LIME explanation 3. **(d)** Original image and LIME explanation 4. **(e)** Original image and LIME explanation 5. **(f)** Original image and LIME explanation 6. **(g)** Original image and LIME explanation 7. **(h)** Original image and LIME explanation 8. **(i)** Original image and LIME explanation 9. **(j)** Original image and LIME explanation 10.

The model incorporates explainability through the use of LIME (Local Interpretable Model-agnostic Explanations) (27) to elucidate its predictions. A custom function, explain_model_predictions, is designed to integrate LIME into the workflow. The process begins with a preprocessing step where each input image is cropped to focus on the region containing the object of interest, achieved using a custom crop_to_object function. Since LIME requires three-channel images, grayscale inputs are converted to RGB format during preprocessing. Once prepared, LIME is applied to generate feature-based explanations for the model's predictions. This involves identifying and highlighting specific parts of the image that influence the model's output. To make these explanations accessible and intuitive, the original cropped image and its LIME-generated explanation are displayed side by side, facilitating easy comparison. This functionality is showcased for up to 10 images selected from the batch loader, providing a detailed explanation of how the model makes decisions. Figure 4 presented the selected images.

The model is trained over a specified number of epochs, with several key steps performed in each iteration. At the start of each epoch, the model is set to training mode, enabling weight updates during the training process. Each batch of images is then processed iteratively, where the loss is calculated using the CrossEntropyLoss function, and the optimizer performs backpropagation followed by parameter updates. The running loss is monitored throughout this procedure, and the model's predictions are compared to the ground-truth labels to calculate the training accuracy. The average loss and accuracy are recorded, printed, and saved for later examination at the conclusion of each epoch. Through the reported losses and accuracies, this methodical technique enables the tracking of the model's performance trends, offering important insights into its learning progress across several epochs.

### 3.4 Inverted residual self-attention CNN model

**Figure 5:**
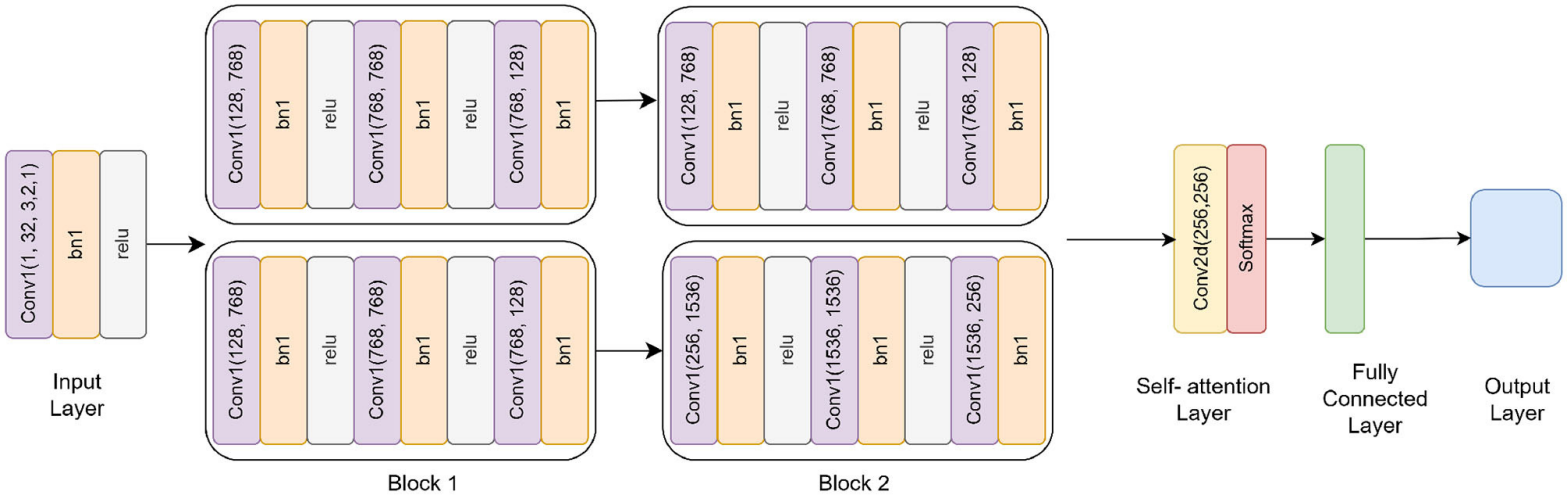
Inverted residual self-attention CNN model architecture.

The Inverted Residual with Self-Attention (IRbACNN) model introduces an innovative design by leveraging inverted residual blocks combined with self-attention mechanisms. This architecture emphasizes efficient feature extraction while maintaining spatial dependencies, making it highly effective for image classification tasks (see Figure 5). The inverted structure focuses on reducing computational overhead by first expanding feature dimensions and then compressing them, resulting in a compact and high-performing model (28).

#### 3.4.1 Input layer and initial convolutional

The IRbACNN model incorporates three inverted residual blocks, each following a consistent structure but with varying input and output channel dimensions. Unlike traditional blocks, the inverted blocks begin by expanding feature dimensions before compressing them, allowing the network to learn high-dimensional intermediate features while reducing the overall parameter count.

**Block 1:** In the first block, the input feature dimensions are expanded from 32 to 128 using a 1 × 1 convolution. This initial expansion increases the representational capacity of the model while keeping computational overhead minimal. Following this, a 3 × 3 depthwise convolution is applied, which preserves the spatial dimensions of the input while significantly reducing computational complexity. To ensure efficient representation, the features are then compressed back to 32 channels using another 1 × 1 convolution. After each convolutional operation, batch normalization is performed to stabilize the training process, and ReLU activation introduces non-linearity, enabling the model to capture complex patterns.

**Block 2:** The second block builds on the structure of the first block, adapting it to process higher-dimensional features. Here, the feature dimensions are expanded from 32 to 256 using a 1 × 1 convolution. This expansion allows the network to extract richer and more intricate patterns from the data. After the depthwise convolution, the features are compressed back to 64 channels, balancing feature richness with computational efficiency. The increased feature depth at this stage bridges the gap between low-level features captured earlier in the network and high-level abstractions learned in later stages.

**Block 3:** In the final block, the feature dimensions undergo the most significant expansion, increasing from 64 to 512 using a 1 × 1 convolution. This stage is designed to capture highly abstract and fine-grained features that are critical for distinguishing between subtle variations in the input data. After the depthwise convolution, the features are compressed back to 128 channels, ensuring compact and efficient representation. This block is essential to the model's capacity to recognize discriminative patterns necessary for complex classification tasks.

#### 3.4.2 Self-attention mechanism

The self-attention mechanism in the IRbACNN model plays an important role in enhancing spatial dependencies and emphasizing discriminative regions of the input. It consists of:

A 1 × 1 convolution to reduce feature dimensionality for computational efficiency. A softmax activation along spatial dimensions to compute an attention map. Element-wise multiplication of the attention map with the feature map, amplifying important features while suppressing irrelevant ones. This mechanism allows the model to focus dynamically on critical regions of the input image, improving its classification performance.

#### 3.4.3 Dynamic fully connected layer

Similar to the RbACNN model, the IRbACNN employs a dynamically initialized fully connected (FC) layer. After applying the self-attention mechanism, the feature map is flattened into a 1D vector. The FC layer adapts to the number of classes in the dataset, ensuring the model's scalability across diverse tasks.

#### 3.4.4 Model training and optimization

With an initial learning rate of 0.001, the Adam optimizer and the cross-entropy loss function are used to optimize the IRbACNN model. Important training actions consist of:

Forward propagation through the model to compute predictions. Loss calculation using CrossEntropyLoss for multi-class classification. Backpropagation to update weights. Each training epoch records and logs the average loss and accuracy, allowing for performance monitoring and evaluation over time.

#### 3.4.5 Explainability with LIME

To enhance interpretability, the IRbACNN model integrates Local Interpretable Model-agnostic Explanations (LIME). The explainability process involves the following:

Preprocessing input images by cropping to the region of interest and converting grayscale images to RGB if necessary. Generating feature-based explanations using LIME to highlight image regions influencing the model's predictions. Visualizing the original image alongside its LIME explanation for comparison and insight into the model's decision-making. This approach improves transparency and trust in the model by providing clear visual explanations for its predictions.

**Figure 6:**
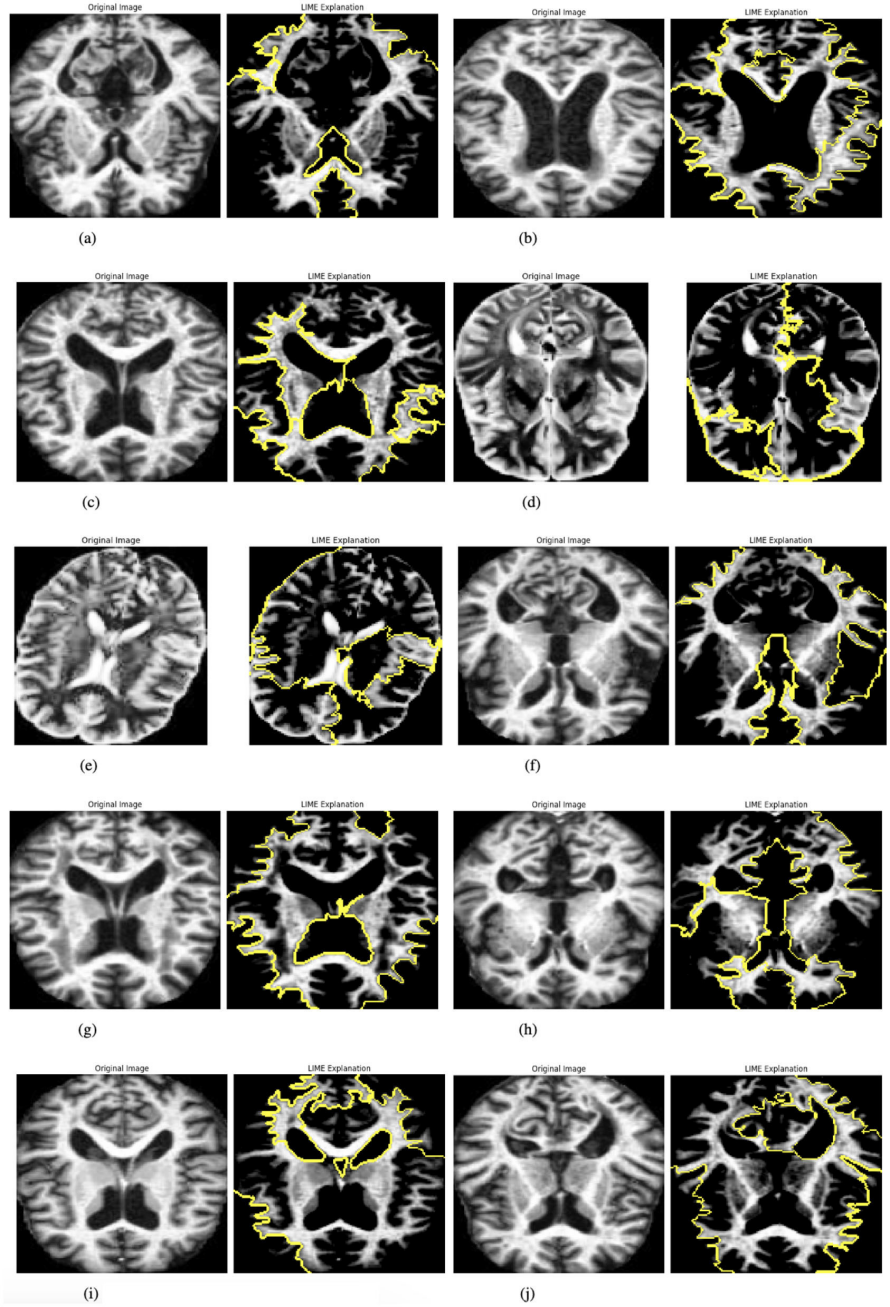
Graphical visualization of original image and explainability LIME Of IRbACNN model. **(a)** Original image and LIME explanation 1. **(b)** Original image and LIME explanation 2. **(c)** Original image and LIME explanation 3. **(d)** Original image and LIME explanation 4. **(e)** Original image and LIME explanation 5. **(f)** Original image and LIME explanation 6. **(g)** Original image and LIME explanation 7. **(h)** Original image and LIME explanation 8. **(i)** Original image and LIME explanation 9. **(j)** Original image and LIME explanation 10.

Once prepared, LIME is applied to generate feature-based explanations for the model's predictions. This involves identifying and highlighting specific parts of the image that influence the model's output. To make these explanations accessible and intuitive, the original cropped image and its LIME-generated explanation are displayed side by side, facilitating easy comparison. This functionality is showcased for up to 10 images selected from the batch loader, providing a detailed explanation of how the model makes decisions. Figure 6 presented the selected images.

The model is trained over a specified number of epochs, with several key steps performed in each iteration. At the start of each epoch, the model is set to training mode, enabling weight updates during the training process. Each batch of images is then processed iteratively, where the loss is calculated using the CrossEntropyLoss function, and the optimizer performs backpropagation followed by parameter updates. The running loss is monitored throughout this procedure, and the model's predictions are compared to the ground-truth labels to calculate the training accuracy. The average loss and accuracy are recorded, printed, and saved for later examination at the conclusion of each epoch. Through the reported losses and accuracies, this methodical technique enables the tracking of the model's performance trends, offering important insights into its learning progress across several epochs.

## 4 Results and discussion

This Section explains the evaluation metrics, experimental findings, and conclusions, including suggestions for future research. The performance of the models was evaluated using the following metrics:

**Accuracy:** This measures the efficiency of the model by showing the ratio of total instances separated correctly while normal and anomalous to the total instances. The total score is an estimate of the model's capacity to generalize normal and abnormal driving styles.

**Loss:** There is also a measure of loss, the Mean Squared Error (MSE), that calculates the average height of the squared vertical distances between the two lines or between the line and the target points. Therefore, a lower loss is preferred since it means the model can effectively reconstruct the initial input data.

### 4.1 Result of the models

Table 3 presents the training performance of a model over 10 epochs. The results show a consistent reduction in loss and a steady improvement in accuracy as training progresses. The initial training loss is 0.7827, with an accuracy of 67.54%, indicating the model's initial performance. Over subsequent epochs, the loss decreases significantly, reaching 0.0379 by the 10(*th*) epoch, while accuracy improves to a peak of 98.83%. This trend demonstrates that the model is effectively learning the data patterns within the given 10 epochs, achieving high accuracy and a low loss value in a relatively short training duration.

**Table 3:** Training loss and accuracy over 10 epochs.

**Epoch**	**Loss**	**Accuracy (%)**
1	0.7827	67.54
2	0.5327	75.79
3	0.4058	82.38
4	0.3028	87.17
5	0.2230	91.29
6	0.1647	93.29
7	0.0900	96.88
8	0.0595	98.17
9	0.0428	98.29
10	0.0379	98.83

Table 4 details the training metrics of a model trained for 25 epochs. Starting with a loss of 0.5265 and an accuracy of 78.42%, the model shows rapid improvement in the early epochs. By epoch 9(*th*), the accuracy reaches 95.25% with a loss of 0.1496. However, the training process exhibits some fluctuations in the later epochs, such as between epochs 19(*th*) and 20(*th*), where accuracy slightly decreases from 94.96% to 94.33%, possibly due to overfitting or data variability. Despite these fluctuations, the model achieves its best results at epoch 25(*th*), with a final loss of 0.0670 and an accuracy of 98.04%. This extended training period provides insights into the model's performance stability and refinement over more iterations.

**Table 4:** Training loss and accuracy over 25 epochs.

**Epoch**	**Loss**	**Accuracy (%)**
1	0.5265	78.42
2	0.4008	84.71
3	0.3574	86.62
4	0.3147	88.25
5	0.2508	90.38
6	0.2394	90.17
7	0.2323	91.83
8	0.2165	92.25
9	0.1496	95.25
10	0.1556	94.88
11	0.1535	94.17
12	0.1624	94.29
13	0.1345	95.50
14	0.1288	95.54
15	0.1278	95.88
16	0.1358	95.62
17	0.1205	96.17
18	0.1205	96.33
19	0.1457	94.96
20	0.1713	94.33
21	0.0947	97.17
22	0.1030	96.71
23	0.1345	96.12
24	0.1025	96.92
25	0.0670	98.04

**Figure 7:**
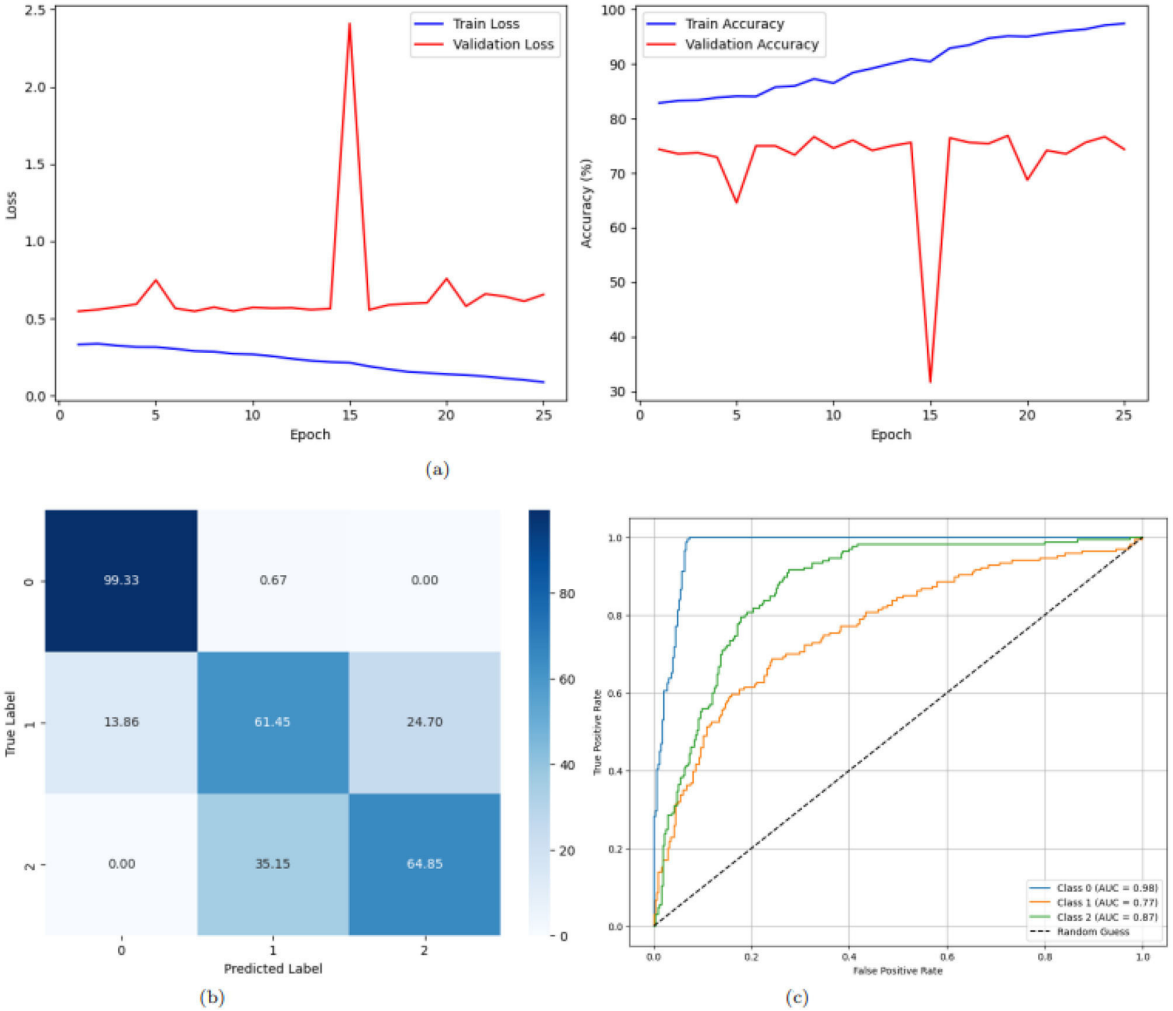
Graphical representation of the model. **(a)** Accuracy and loss of the model. **(b)** CM of the model. **(c)** ROC of the model.

Figure 7 illustrates the training and validation loss and accuracy trends, confusion matrix, and ROC curves for a deep learning model applied to a multi-class classification task with three classes: AD, PD, and CONTROL. Figure 7a illustrates the training and validation loss and accuracy trends over 25 epochs for a deep learning model applied to multi-classification tasks. The left graph shows the loss curve, while the right graph represents the accuracy curve. The Training Loss curve indicates a gradual reduction in loss values as the number of epochs increases. Starting at approximately 0.8 at the 1(*st*) epoch, the loss consistently decreases with each epoch and stabilizes around 0.1 after the 20(*th*) epoch. This steady decline and eventual plateau suggest that the model effectively minimizes the error over time, indicating successful learning. The Training Accuracy curve starts at around 70% for validation and 80% for training; the validation curves stabilize, reaching up to 74% Meanwhile, the training curve rapidly increases over the first few epochs, reaching nearly 97% by the 23(*rd*) epoch. The accuracy then stabilizes, showing minimal fluctuations, and remains consistently high. This pattern shows that the model performs very well on the training set by successfully capturing the patterns in the training data. Figure 7b presents the confusion matrix for the multi-class classification task. The matrix visualizes the true labels versus the predicted labels, providing insights into the model's performance for each class. The diagonal cells represent correct classifications, with the values being relatively high for all three classes (Class 0, Class 1, and Class 2). For instance, 99.33% instances of Class 0, 61.45% instances of Class 1, and 64.85% instances of Class 2 were correctly classified. The off-diagonal cells highlight misclassifications. For Class 1, 24.70% samples were incorrectly classified as Class 2. Similarly, Class 2 had 35.15% samples misclassified as Class 1. The confusion matrix demonstrates that the model performs well overall, with misclassifications for class 2, indicating robust classification performance. Figure 7c depicts the ROC curves for a multi-class classification task. Every line depicts the ROC curve of each class to measure the TPR against FPR optimally. The AUC values are different for all three classes, Class 0 with 0.98, class 1 with 0.77, and Class 2 with 0.87, which indicates that the classes can be discriminated at a good rate. The curves are right on top of each other, and they stay very close to the top-left corner, which proves the model's discriminative strengths. Altogether, the results confirm the high efficiency of the model in distinguishing between the classes and the nearly perfect classification accuracy for all the classes.

**Figure 8:**
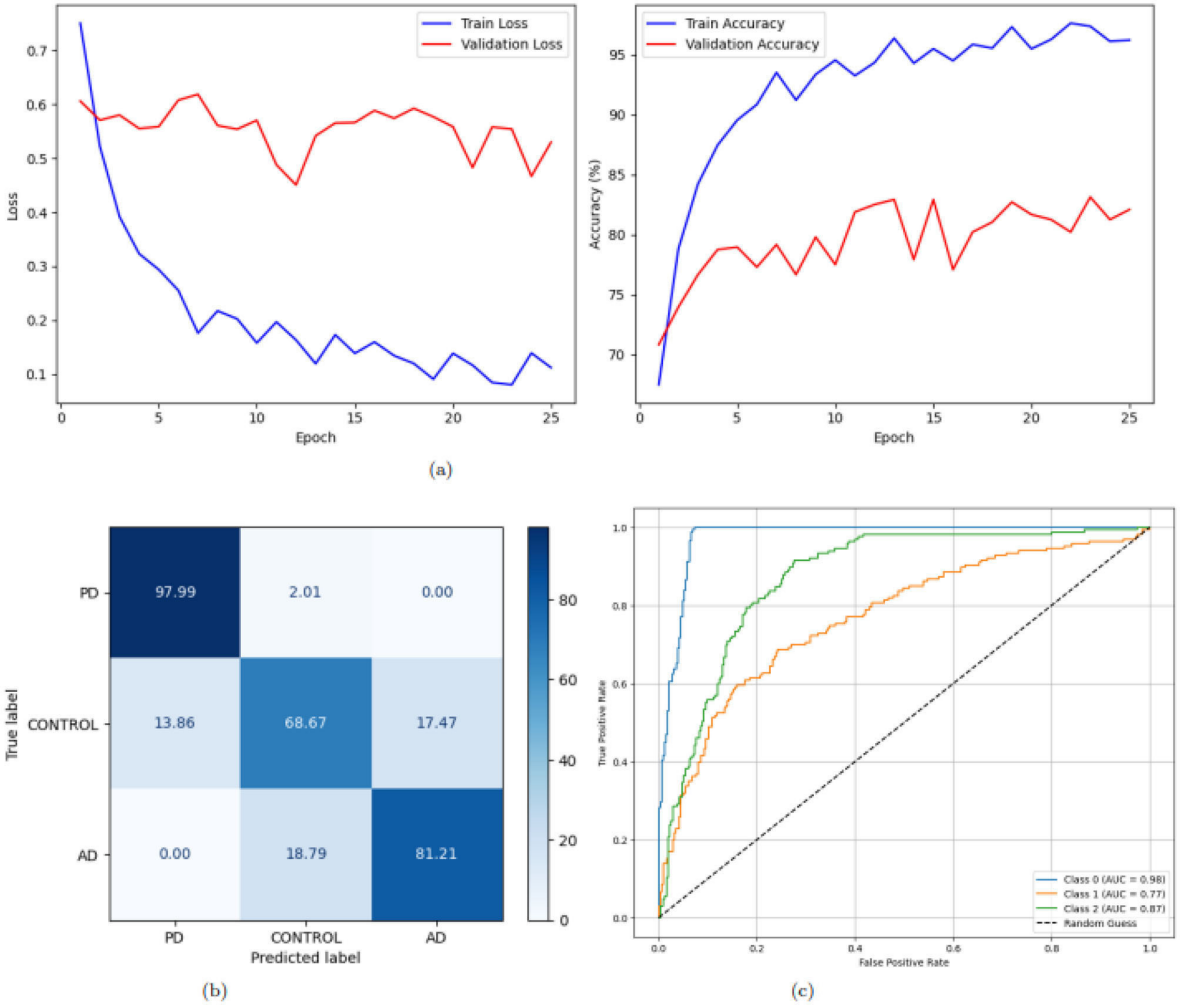
Graphical representation of the model. **(a)** Accuracy and loss of the model. **(b)** CM of the model. **(c)** ROC of the model.

Figure 8 illustrates the training loss and accuracy trends, confusion matrix, and ROC curves for a deep learning model applied to a multi-class classification task with three classes: AD, PD, and CONTROL. The training loss curve, as shown in Figure 8a on the left, exhibits a consistent reduction in loss values over 25 epochs. Beginning at approximately 0.75 for training and 0.6 for validation, the loss decreases steadily, dropping below 0.2 by the 7(*th*) epoch and stabilizing around 0.1 by the 25(*th*) epoch for training and validation it dropped below 0.5. This steady decline and eventual stabilization suggest effective learning and convergence of the model. The training accuracy curve, shown on the right, starts at approximately 66% for training and 71% for validation and improves rapidly, surpassing 90% for training and 80% for validation within the first 10(*th*) epochs demonstrating the model's strong ability to learn patterns from the training data and achieve high accuracy. The confusion matrix in Figure 8b gives the classification performance of the model with more clarity. The diagonal values, representing correctly classified instances, are notably high for class 0 and class 2: 97.99% samples correctly classified as PD and 97.99% as PD but only 68.67% as CONTROL. Figure 8c presents the ROC curves for the three classes. Every line depicts the ROC curve of each class to measure the TPR against FPR optimally. The AUC values are 0.98, 0.87 and 0.96 for class 0, class 1 and class 2, respectively. The ROC curves are in proximity to the top-left corner of the graph, which means that the model is a great classifier. In conclusion, the general outlines of the training loss and accuracy make us believe that, in general, the model excels in multi-class classification.

### 4.2 Comparison and discussion

Table 5 presents a comparative analysis of recent studies on Alzheimer's and Parkinson's Disease classification using machine learning and deep learning techniques. Various models have been explored, each leveraging different architectures and preprocessing strategies to improve classification performance. Authors in (29) introduced a deep hybrid network combining ensemble classifiers with CNNs and utilized a super-resolution neural network for MRI preprocessing, achieving an accuracy of 99.11% on a three-class MRI dataset. Similarly, Siddiqua et al. (30) investigated transfer learning using four CNN architectures–EfficientNetB0, ResNet50, InceptionV3, and Xception–on a three-class MRI dataset, with EfficientNetB0 outperforming others with an accuracy of 99.4%. Meanwhile, Nancy Noella and Priyadarshini (31) applied classical machine learning classifiers, including Bagged Ensemble, ID3, Naive Bayes, and Multi-class SVMs, on PET images. The Bagged Ensemble classifier achieved the highest accuracy of 90.3% on a dataset of 1,050 images. Our proposed approach builds upon these advancements by introducing enhanced deep learning architectures, RbACNN and IRbACNN, which integrate self-attention mechanisms to enhance feature extraction. Additionally, histogram equalization is employed as a preprocessing step to improve contrast and enhance model performance. Our framework achieves an accuracy of 99.92% on publicly available datasets containing AD, PD, and normal brain images. Beyond accuracy, our findings highlight several key aspects of deep learning applications in neurodegenerative disease classification. First, self-attention mechanisms improve model interpretability by focusing on the most relevant image features. Second, preprocessing techniques such as histogram equalization significantly enhance image quality, leading to more robust feature extraction. Lastly, while traditional ML methods still offer reasonable performance, deep learning approaches–particularly with attention mechanisms–demonstrate superior accuracy and generalization capabilities. Despite these advantages, challenges remain. One limitation of deep learning models is their reliance on large datasets to generalize effectively. Additionally, the “black-box” nature of some architectures can hinder clinical adoption due to a lack of explainability. Future research should focus on integrating explainable AI techniques to enhance model transparency and developing strategies to address data scarcity, such as data augmentation and synthetic data generation. In summary, our proposed models outperform existing approaches in terms of accuracy while addressing critical limitations in neurodegenerative disease classification. These findings underscore the potential of deep learning in advancing automated diagnostic tools for AD and PD, ultimately aiding early detection and improving patient outcomes.

**Table 5:** Comparison with related work.

**References**	**Method/approach**	**Dataset**	**Performance metrics**
Alhudhaif (29)	Hybrid CNN with ensemble classification; super-resolution preprocessing for MRI	Public 3-class MRI dataset (AD, PD, Healthy)	Accuracy: 99.11%
Siddiqua et al. (30)	Transfer learning with EfficientNetB0, ResNet50, InceptionV3, Xception	3-class MRI dataset (AD, PD, Healthy)	Best: EfficientNetB0, Accuracy: 99.4%
Nancy Noella and Priyadarshini (31)	ML classifiers (Bagged Ensemble, ID3, Naive Bayes, SVM) on PET images	PET dataset (1050 images; AD, PD, Healthy)	Best: Bagged Ensemble, Accuracy: 90.3%
Proposed research	RbACNN and IRbACNN with self-attention; histogram equalization preprocessing	Public datasets (AD, PD, Healthy)	Accuracy: 99.92%

## 5 Conclusion

This study effectively presented two deep learning frameworks, RbACNN and IRbACNN, for a multi-class classification of Alzheimer's Disease and Parkinson's Disease. Through the incorporation of self-attention mechanisms, these models refine the ability to perform feature extraction, leading to better performance and interpretability in scenarios using medical images. The steps of preprocessing that have been used are histogram equalization and the creation of batches in order to enhance the performance of the model. The results obtained from experiments suggest that the proposed models can classify AD, PD, and control accurately with a classification rate of 99.92%. Based on the findings, further practical implications are suggested toward encouraging the use of deep learning techniques in detecting neurodegenerative diseases during the early stages so as to enable early intervention and management. In the future, the researcher will push their current work even more on how to enhance these models and how to apply them to the biggest datasets, including different types of data. Furthermore, applying explainability techniques, including LIME, will be extended to increase the acceptability of AI in clinical practice. Finally, the developments discussed in this research aim to advance AI-based approaches in the battle against neurodegenerative diseases to provide better diagnostics and better patient outcomes.

## Data Availability

The original contributions presented in the study are included in the article/supplementary material, further inquiries can be directed to the corresponding authors.
